# Stimulus–effect relations for left ventricular growth obtained with a simple multi-scale model: the influence of hemodynamic feedback

**DOI:** 10.1007/s10237-020-01327-2

**Published:** 2020-05-01

**Authors:** Emanuele Rondanina, Peter H. M. Bovendeerd

**Affiliations:** Technische Universiteit Eindhoven, PO Box 513, 5600 MB Eindhoven, The Netherlands

**Keywords:** Hemodynamic feedback, Cardiac growth, Left ventricle, Growth stimuli

## Abstract

Cardiac growth is an important mechanism for the human body to respond to changes in blood flow demand. Being able to predict the development of chronic growth is clinically relevant, but so far models to predict growth have not reached consensus on the stimulus–effect relation. In a previously published study, we modeled cardiac and hemodynamic function through a lumped parameter approach. We evaluated cardiac growth in response to valve disease using various stimulus–effect relations and observed an unphysiological decline pump function. Here we extend that model with a model of hemodynamic feedback that maintains mean arterial pressure and cardiac output through adaptation of peripheral resistance and circulatory unstressed volume. With the combined model, we obtain stable growth and restoration of pump function for most growth laws. We conclude that a mixed combination of stress and strain stimuli to drive cardiac growth is most promising since it (1) reproduces clinical observations on cardiac growth well, (2) requires only a small, clinically realistic adaptation of the properties of the circulatory system and (3) is robust in the sense that results were fairly insensitive to the exact choice of the chosen mechanics loading measure. This finding may be used to guide the choice of growth laws in more complex finite element models of cardiac growth, suitable for predicting the response to spatially varying changes in tissue load. Eventually, the current model may form a basis for a tool to predict patient-specific growth in response to spatially homogeneous changes in tissue load, since it is computationally inexpensive.

## Introduction

The capability of the human body to maintain an adequate level of oxygen delivery to the organs is fundamental for survival. The body can rely on several complex mechanisms to achieve this goal. Cardiac growth is the main mechanism of response to chronic changes in blood flow demand, induced for example in the growing body. An in depth review of the cardiovascular adaptations from fetus to adolescence can be found in Dallaire and Sarkola (2018). Cardiac growth, although essential, can evolve into a maladaptive process if the growth stimulus is severe or brusquely applied, leading to a pathological type of growth (Grossman 1980). A disease capable of altering either the preload or afterload of the cardiovascular system, like for instance any valve disease, can promote an abnormal type of growth. Left ventricular hypertrophy has been related to an adverse prognosis during long-term follow-ups, increasing the chance of mortality (Gosse 2005; Muiesan et al. 2004; Pierdomenico et al. 2011; Selmeryd et al. 2014; Spirito et al. 2000; Tuseth et al. 2010). Moreover, although cardiac growth phenotypes are well characterized (Dweck et al. 2012; Ganau et al. 1992; Rodrigues et al. 2016), the relation between the growth stimulus and the long-term effects on the cardiovascular system is still not completely clear. Being able to predict changes in left ventricular size and shape not only will increase the knowledge on cardiac growth, but it might also help patient prognosis and guide the treatment of choice.

So far several models of cardiac growth (Arts et al. 2005; Göktepe et al. 2010; Humphrey and Rajagopal 2002; Kerckhoffs et al. 2012b; Kroon et al. 2009; Lin and Taber 1995; Taber 1998) have been proposed, along with reviews on the state of the art (Bovendeerd 2012; Witzenburg and Holmes 2017); however, the nature of the growth stimulus is still under debate. In a recent paper (Rondanina and Bovendeerd 2020), we studied growth of the left ventricle (LV) using a simple multiscale model. We designed a growth law capable of coupling changes in tissue mechanical load, identified as growth stimuli, into a volumetric change, expressed by LV wall and cavity volume. We explored several choices and combinations of growth stimuli, both stress based and strain based, with the aim to investigate the stimulus–effect relation. We investigated growth in response to three cases of valve disease, aortic stenosis (AS), aortic regurgitation (AR) and mitral regurgitation (MR). Although we were able to achieve stable end growth states, in most cases we obtained a drastic decrease in cardiac output (CO) and mean arterial pressure (MAP) between 20 and 40%. Even though valve pathologies might decrease cardiac function (Goodman et al. 1974; Kamperidis et al. 2015), there is evidence that mean arterial pressure and cardiac output can be maintained at a normal level (Cowley Jr 1992; Guyton 1967; Kainuma et al. 2011; Lorsomradee et al. 2007). If we accept as healthy a cardiac index of about 2.9 l/min/$\hbox {m}^{2}$ (Ganau et al. 1992; Huang et al. 2011; Wisenbaugh et al. 1984) and a MAP of 100 mmHg (Remmen et al. 2005; Rongen et al. 1995), these values are often within the reported ranges for patients having AS (Lloyd et al. 2017; Rajani et al. 2010), AR (Greenberg et al. 1981; Röthlisberger et al. 1993) or MR (Kainuma et al. 2011). However, some studies report a clear decrease in the hemodynamic function (Goodman et al. 1974; Kamperidis et al. 2015; Martinez et al. 2012; Wisenbaugh et al. 1984). This might be caused by an incomplete hemodynamic feedback or by the incapability of the body to cope the disease severity.

In this study, we aim to extend our previous model of cardiac growth with a hemodynamic feedback mechanism which acts upon the circulatory system in order to restore homeostatic levels of pressure and flow. Such mechanisms are known to act on the short term and the long term (Dampney et al. 2002; Hall 2015). Short-term regulation includes feedback processes which can be triggered rapidly, with baroreceptors (Kirchheim 1976), chemoreceptors (Guyenet and Koshiya 1995) and humoral responses (Goodwin et al. 1972; Hilton 1975). The baroreflex feedback is an important short-term mechanism, through which cardiac properties (contractility, heart rate) and vascular properties (peripheral resistance, venous tone) are adapted to maintain mean arterial pressure (Folkow 1978; Guyton 1981; Secomb and Pries 2011). Fluid exchange between the vascular and interstitial space, driven by hemodynamic and osmotic pressure, in combination with neurohumoral control of renal function is known to control vascular volume on the time scale of hours to days. On an even longer timescale, cardiac adaptation in terms of contractility is taken over by growth, while heart rate remains normal (Akinboboye et al. 2004; Ganau et al. 1992; Seldrum et al. 2018). Vascular adaptation is realized through persistent changes in stressed blood volume and systemic vascular resistance (Cowley Jr 1992; Guyton 1981; Jacobsohn et al. 1997; Secomb and Pries 2011).

In line with the approach in our previous work (Rondanina and Bovendeerd 2020), we aim for a phenomenological description of the cardiovascular system adaptations on the long term. We follow previous studies which suggest how vasculature resistance and blood volume can be adapted to regulate the mean arterial pressure (MAP) (Cowley Jr 1992; Guyton 1981; Osborn 2005) and cardiac output (CO) (Guyton et al. 1955; Jacobsohn et al. 1997). CO is an important determinant of the amount of oxygen supplied to the vital organs, while the MAP is the driving force behind CO. Our model aims to recover the CO by updating the afterload of the system, represented by the systemic resistance, while MAP is restored with a change in the preload, described by the stressed blood volume.

The scope of this study is to reevaluate the relation between a growth stimulus and its effects at organ and tissue levels in the presence of the hemodynamic feedback. As in our previous study, we test the model in case of three valve diseases: AS, AR and MR. We evaluate the obtained growth in terms of the left ventricular end diastolic volume index (EDVI), left ventricular mass index (MI) and relative wall thickness (RWT).

## Methods

In this work, we extend the approach proposed in Rondanina and Bovendeerd (2020). More specifically, we extend the three submodels for left ventricular (LV) mechanics, systemic circulation and cardiac growth with a fourth submodel for hemodynamic feedback.

### Left ventricular mechanics model

To describe left ventricular mechanics, we use the one-fiber model (Arts et al. 1991; Bovendeerd et al. 2006) which couples the mechanics at the organ level, identified by left ventricular cavity pressure $p_{\rm {cav}}$ and cavity volume $V_{\rm {cav}}$, with the mechanics at tissue level, described with myofiber stress $\sigma _{\rm {f}}$ and sarcomere length $l_{\rm {s}}$.

1a $$\begin{aligned} p_{\rm {cav}} \;&=\; \frac{1}{3} \; \sigma _{\rm {f}} \; \text {ln}\left( 1 \;+\; \frac{V_{\rm {wall}}}{V_{\rm {cav}}} \right) \end{aligned}$$

1b $$\begin{aligned} \lambda _{\rm {f}} \;&=\; \frac{l_{\rm {s}}}{l_{\rm {s,0}}} \;=\; \left( \frac{V_{\rm {cav}} \;+\; \frac{1}{3} \; V_{\rm {wall}}}{V_{\rm {cav,0}} \;+\; \frac{1}{3} \; V_{\rm {wall}}} \right) ^{\frac{1}{3}} \end{aligned}$$

here $l_{\rm {s,0}}$ is the sarcomere length at zero pressure, $V_{\rm {cav,0}}$ represents the unstressed cavity volume, $V_{\rm {wall}}$ represents the wall volume and $\lambda _{\rm {f}}$ is the fiber stretch ratio. Myofiber stress $\sigma _{\rm {f}}$ consists of an active component, which depends on $l_{\rm {s}}$ and the time elapsed after activation, and a passive component, which depends on $\lambda _{\rm {f}}$. A full description of the model can be found in Bovendeerd et al. (2006) and Rondanina and Bovendeerd (2020).

### Systemic circulation model

**Fig. 1:**
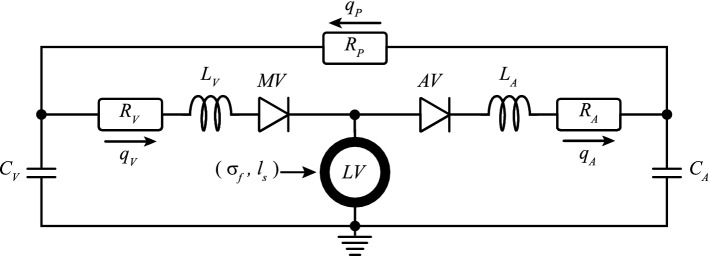
Lumped parameter model of the circulation. With mitral valve (MV), aortic valve (AV), venous and arterial resistance ($R_{\rm {V}}$ and $R_{\rm {A}}$), compliance ($C_{\rm {V}}$ and $C_{\rm {A}}$) and inertance ($L_{\rm {V}}$ and $L_{\rm {A}}$) , peripheral resistance ($R_{\rm {P}}$) and venous, arterial and peripheral flows ($q_{\rm {V}}$, $q_{\rm {A}}$, $q_{\rm {P}}$). This model is coupled with the one-fiber model of left ventricular (LV) mechanics from which we obtain myofiber stress ($\sigma _{\rm {f}}$) and sarcomere length ($l_{\rm {s}}$)

The systemic circulation is described by a lumped parameter model (Fig. 1) which interacts with the LV mechanics model. The arteries (A) and the veins (V) are modeled by a resistance *R*, a capacitor *C* and an inertance *L* in series while the peripheral vessels are approximated by only one resistance. The pressure drop $\varDelta p$ over each resistance, capacitor and inertance is defined as follows:

2a $$\begin{aligned} \varDelta p_{R} \;&=\; R q_{R} \end{aligned}$$

2b $$\begin{aligned} \varDelta p_{C} \;&=\; \frac{V_{C} - V_{C,0}}{C} \end{aligned}$$

2c $$\begin{aligned} \varDelta p_{L} \;&=\; L \frac{\text {d}q_{L}}{\text {d}t} \end{aligned}$$

where *q* is the flow through each segment (*R*, and *L*) while $V_{C}$ and $V_{C,0}$ are the stressed and unstressed volumes that a vessel can accommodate. According to Eqs. 2a and 2c, we can write the arterial flow $q_{\rm {A}}$ as:

3 $$\begin{aligned} p_{\rm {LV}} \;-\; p_{\rm {A}} \;=\; k_{\rm {AV}}\, R_{\rm {A}}\, q_{\rm {A}} \;+\; L_{\rm {A}} \frac{\text {d}q_{\rm {A}}}{\text {d}t} \end{aligned}$$

and the venous flow $q_{V}$ as:

4 $$\begin{aligned} p_{\rm {V}} \;-\; p_{\rm {LV}} \;=\; k_{\rm {MV}}\, R_{\rm {V}}\, q_{\rm {V}} \;+\; L_{\rm {V}} \frac{\text {d}q_{\rm {V}}}{\text {d}t} \end{aligned}$$

where $p_{\rm {LV}}$ is the LV cavity pressure, $p_{\rm {A}}$ and $p_{\rm {V}}$ are the arterial and venous pressure, $R_{\rm {A}}$ and $R_{\rm {V}}$ are the arterial and venous resistance, and $L_{\rm {A}}$ and $L_{\rm {V}}$ are the arterial and venous inertance, respectively. The aortic valve (AV) and mitral valve (MV) are approximated as a diode which function is regulated by the parameters $k_{\rm {AV}}$ and $k_{\rm {MV}}$. For an healthy AV, $k_{\rm {AV}}$ is equal to 1 when $p_{\rm {LV}}$ is higher than $p_{\rm {A}}$; otherwise, it has a value of $10^{6}$. Similarly for an healthy MV, $k_{\rm {MV}}$ is equal to 1 when $p_{\rm {V}}$ is higher than $p_{\rm {LV}}$; otherwise, it has a value of $10^{6}$. The peripheral flow $q_{\rm {P}}$ is described with Eq. 2a as follows:

5 $$\begin{aligned} q_{\rm {P}} \;=\; \frac{p_{\rm {A}} - p_{\rm {V}}}{R_{\rm {P}}} \end{aligned}$$

where $R_{\rm {P}}$ represents the resistance generated by all the peripheral vessels. Moreover, we compute the cardiac output (CO) as the average of $q_{\rm {P}}$ over a complete cardiac cycle.

Pressure levels in the model are dependent on the total stressed blood volume $V_\mathrm{{sb}}$, that identifies the amount of blood volume exceeding the sum of all unstressed blood volumes:

6 $$\begin{aligned} V_\mathrm{{sb}} = V_{\rm {tot}} \;-\; \sum _{n} V_{n,0} \end{aligned}$$

where the summation of *n* includes the zero pressure volumes of arteries ($V_{\rm {A},0}$), veins ($V_{\rm {V},0}$) and ventricle ($V_{\rm {cav},0}$). Moreover, $V_\mathrm{{sb}}$ is also related to the mean circulatory filling pressure $p_\mathrm{{mc}}$:

7 $$\begin{aligned} p_\mathrm{{mc}} \;\approx \; \frac{V_\mathrm{{sb}}}{C_{\rm {A}} + C_{\rm {V}}} \end{aligned}$$

where we neglected the compliance of the LV. In turn, $p_\mathrm{{mc}}$ is an important determinant of LV filling pressure, and hence, the LV filling volume. An increase in the filling volume will cause an increase in the sarcomere stretch which in turn will increase the sarcomere active stress. With a higher active stress, the ventricle will develop a higher systolic pressure which will eventually increase the MAP and CO.

### Growth model

Based on our previous work (Rondanina and Bovendeerd 2020), we define the growth stimulus to measure a difference in the sarcomere mechanics between the current state and the homeostatic state (hom). A generic growth stimulus $S_{(\sigma ;\epsilon )}$ is designed to be a function of a stress loading measure $L_{(\sigma )}$ or a strain loading measure $L_{(\epsilon )}$.

8 $$\begin{aligned} S_{(\sigma ;\epsilon )} \;=\; \frac{L_{(\sigma ;\epsilon )} \;-\; L_{(\sigma ;\epsilon ),\text {hom}}}{L_{(\sigma ;\epsilon ),\text {hom}}} \end{aligned}$$

We investigate two types of stress stimuli based on the mean (Eq. 9a) and maximum stress (Eq. 9b):

9a $$\begin{aligned} L_{\sigma }^\mathrm{{avg}}&= \frac{1}{T_{\rm {cyc}}} \; \int _0^{T_{\rm {cyc}}} \! \sigma _{\rm {f}}(t) \; \mathrm {d}t \end{aligned}$$

9b $$\begin{aligned} L_{\sigma }^\mathrm{{max}}&= \text {max}\left[ \sigma _{\rm {f}}(t)\right] \end{aligned}$$

where $T_{\rm {cyc}}$ is the cardiac cycle length. As strain stimuli, we consider the sarcomere strain amplitude (Eq. 10a) and the maximum strain (Eq. 10b):

10a $$\begin{aligned} L_{\epsilon }^\mathrm{{amp}}&= \text {max}\left[ \text {ln} \left( \lambda _{\rm {f}}\right) \right] \;-\; \text {min}\left[ \text {ln} \left( \lambda _{\rm {f}}\right) \right] \end{aligned}$$

10b $$\begin{aligned} L_{\epsilon }^\mathrm{{max}}&= \text {max}\left[ \text {ln} \left( \lambda _{\rm {f}}\right) \right] \end{aligned}$$

The growth stimulus is then converted into growth of the wall volume $V_{\rm {wall}}$ and the unstressed cavity volume $V_{\rm {cav,0}}$ according to the following law for stress-based stimuli:

11a $$\begin{aligned} \frac{1}{V_{\rm {wall}}} \; \frac{\text {d}V_{\rm {wall}}}{\text {d}t}&\;=\; +\; \frac{S_{\sigma }}{\tau _{\rm {grw}}} \end{aligned}$$

11b $$\begin{aligned} \frac{1}{V_{\rm {cav,0}}} \; \frac{\text {d}V_{\rm {cav,0}}}{\text {d}t}&\;=\; -\; \frac{S_{\sigma }}{\tau _{\rm {grw}}} \end{aligned}$$

and for strain-based stimuli:

12a $$\begin{aligned} \frac{1}{V_{\rm {wall}}} \; \frac{\text {d}V_{\rm {wall}}}{\text {d}t}&\;=\; +\; \frac{S_{\epsilon }}{\tau _{\rm {grw}}} \end{aligned}$$

12b $$\begin{aligned} \frac{1}{V_{\rm {cav,0}}} \; \frac{\text {d}V_{\rm {cav,0}}}{\text {d}t}&\;=\; +\; \frac{S_{\epsilon }}{\tau _{\rm {grw}}} \end{aligned}$$

where $\tau _{grw}$ is the growth time constant. The sign in Eqs. 11 and 12 is related to the chosen $L_{(\sigma ;\epsilon )}$ and it is defined such that any divergence from the homeostatic state of Eq. 8 is correctly balanced by a change in $V_{\rm {wall}}$ and $V_{\rm {cav,0}}$. The reader might refer to our previous manuscript (Rondanina and Bovendeerd 2020) for an in depth discussion on this model.

The combination of four growth stimuli (Eqs. 9 – 10) and two growth laws (Eqs. 11a and 12a for $V_{\rm {wall}}$, Eqs. 11b and 12b for $V_{\rm {cav,0}}$) results in sixteen possible combinations that can be evaluated, see Table 1. The four cases in which a strain stimulus drives both cavity and wall growth are labeled as ‘strain-only’ cases. Similarly, we identify four ‘stress-only’ cases. The remaining eight cases involve both stress and strain stimuli and are labeled as ‘mixed’ cases. As in our previous study (Rondanina and Bovendeerd 2020), we found that switching the stimuli for cavity and wall growth did not affect the final grown state. Hence, we evaluate only four cases.

**Table 1:**
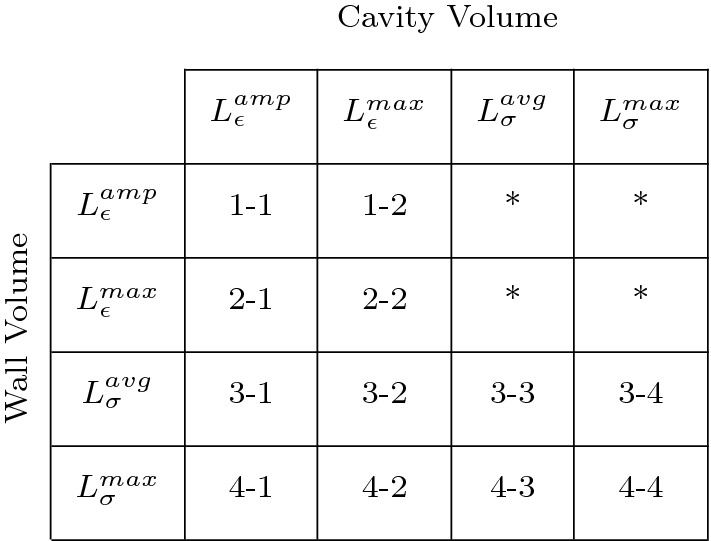
Overview of stimulus–effect relations along with the corresponding label used in Figs. 2, 3, 4, 5, 6 and 7. The asterisks in the upper right corner refer to simulations that are not presented since they show identical end states of growth as the corresponding simulations in the lower left corner, characterized by a switch of stimuli for wall and cavity growth

**Fig. 2:**
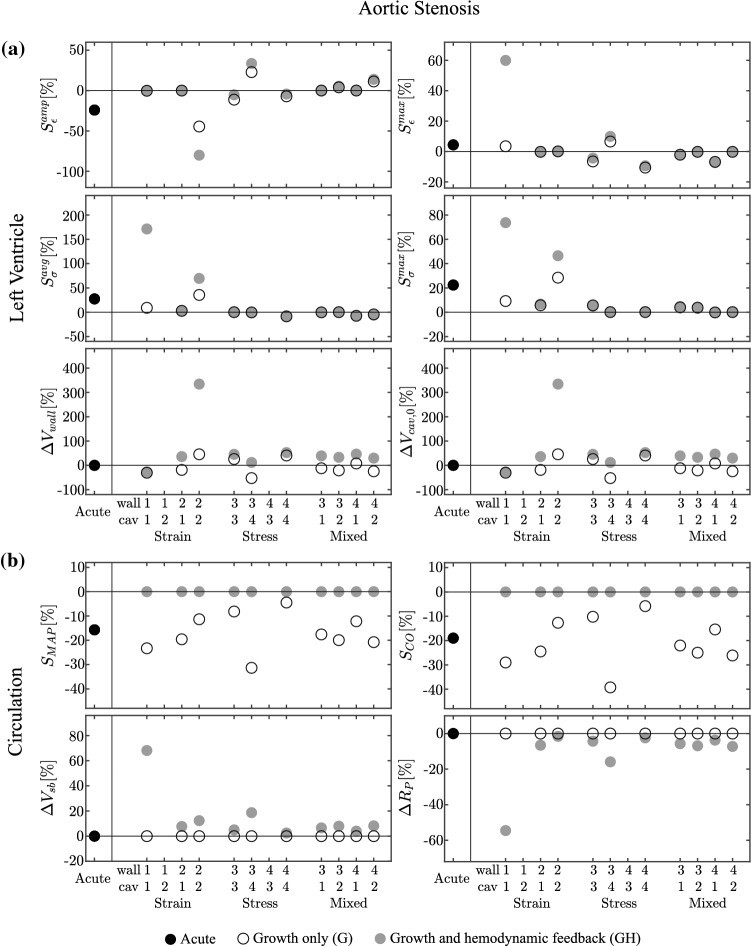
Aortic stenosis (AS) case for the acute state (Acute), the growth only cases (G) and the cases with growth and hemodynamic feedback (GH). Results are grouped by a strain stimulus only, a stress stimulus only, and a mixed stimulus of both stress and strain. For ease of notation, on the horizontal axis the four stimuli are denoted by: (1) sarcomere strain amplitude $S_{\epsilon }^\mathrm{{amp}}$, (2) maximum strain $S_{\epsilon }^\mathrm{{max}}$, (3) average sarcomere stress $S_{\sigma }^\mathrm{{avg}}$ and (4) maximum stress $S_{\sigma }^\mathrm{{max}}$, see also Tab. (1). Panel a) shows changes in wall volume ($\varDelta V_{\rm {wall}}$) and unstressed cavity volume ($\varDelta V_{\rm {cav,0}}$), along with the final values of the four stimuli. Panel b) shows changes in peripheral resistance ($R_{\rm {P}}$) and total stressed blood volume ($V_\mathrm{{sb}}$), along with the final values of the hemodynamic stimuli related to mean arterial pressure ($S_\mathrm{{MAP}}$) and cardiac output ($S_\mathrm{{CO}}$). All the results are shown in respect with the homeostatic state. Eventually cases 1-2 and 4-3 are unstable for both G and GH simulations

**Fig. 3:**
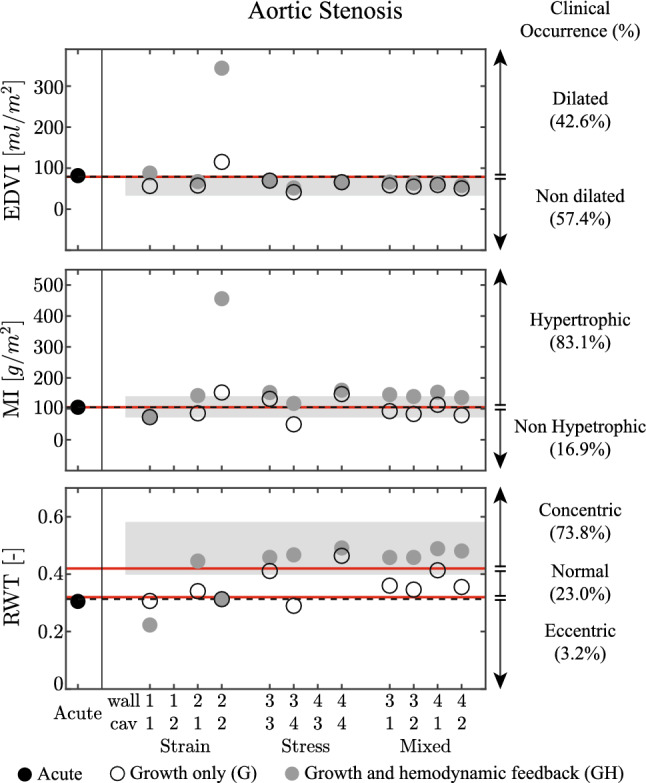
Aortic stenosis (AS) case for the acute state (Acute), the growth only cases (G) and the cases with growth and hemodynamic feedback (GH). Results are grouped by a strain stimulus only, a stress stimulus only, and a mixed stimulus of both stress and strain. For ease of notation, on the horizontal axis the four stimuli are denoted by: (1) sarcomere strain amplitude $S_{\epsilon }^\mathrm{{amp}}$, (2) maximum strain $S_{\epsilon }^\mathrm{{max}}$, (3) average sarcomere stress $S_{\sigma }^\mathrm{{avg}}$ and (4) maximum stress $S_{\sigma }^\mathrm{{max}}$, see also Tab. (1). The figure shows the left ventricular end diastolic volume index (EDVI), mass index (MI) and relative wall thickness (RWT). On the three panels, patient data are presented as mean with standard deviation (Guzzetti et al. 2019), while on the right side patient data are represented as clinical occurrence in percentage (Barbieri et al. 2019a). The left ventricle is considered dilated if EDVI > 79 ml/$\hbox {m}^{2}$, hypertrophic if MI > 105 g/$\hbox {m}^{2}$, and with an eccentric geometry, with RWT < 0.32, normal geometry, with 0.32 $\le$ RWT $\le$ 0.42, and concentric geometry, with RWT > 0.42 (Gaasch and Zile 2011). The dashed lines identify the homeostatic level

**Fig. 4:**
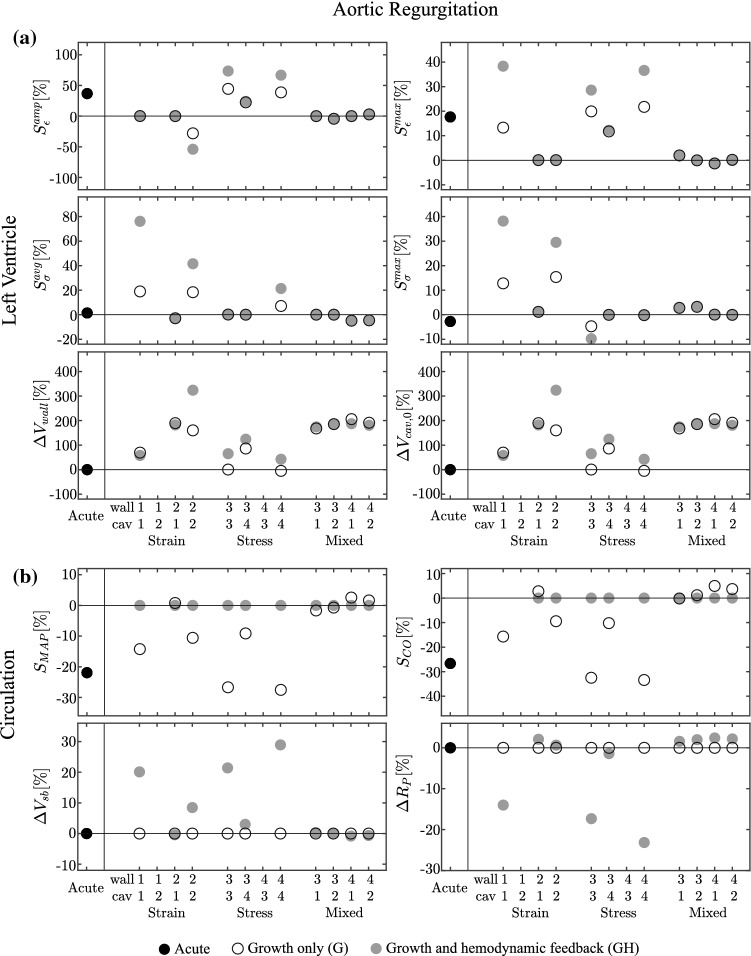
Results for the aortic regurgitation (AR) case, presented according to the format in Fig. 2. Cases 1-2 and 4-3 are unstable for both G and GH simulations

**Fig. 5:**
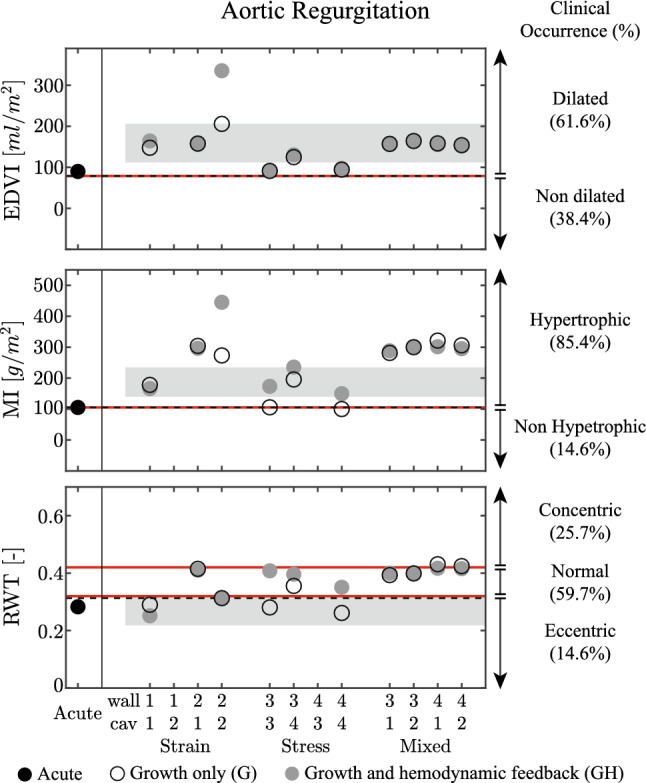
Results for the aortic regurgitation (AR) case, presented according to the format in Fig. 3. On the three panels, patient data are identified as mean with standard deviation, with data collected from Wisenbaugh et al. 1984 for end diastolic volume EDVI and mass index MI while Seldrum et al. (2018) is used for relative wall thickness RWT. On the right side, patient data are represented as clinical occurrence in percentage (Barbieri et al. 2019b)

**Fig. 6:**
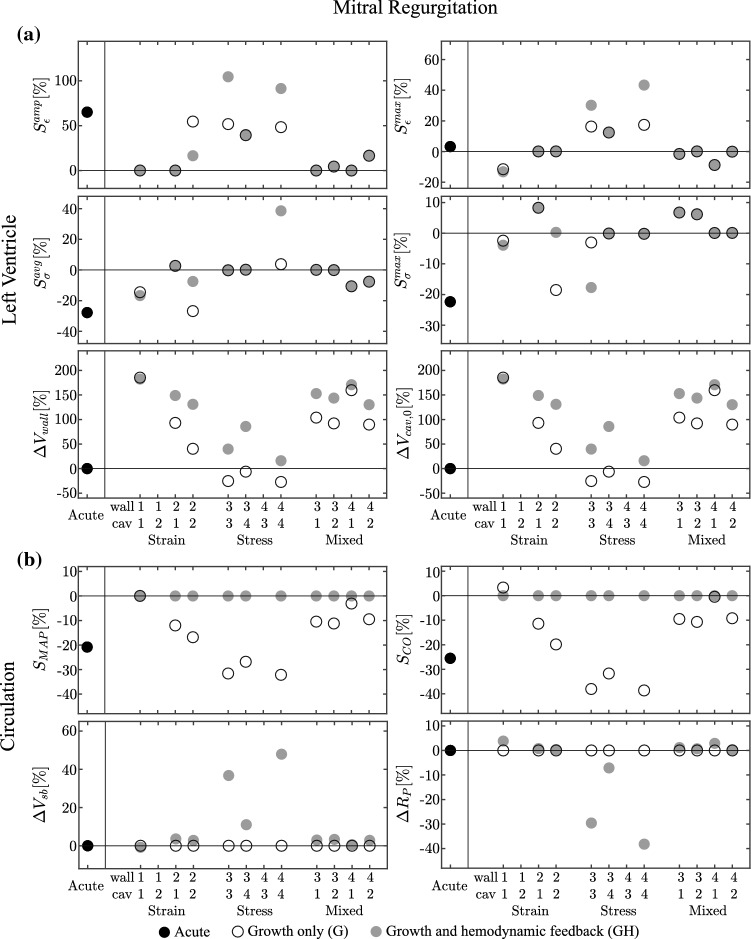
Results for the mitral regurgitation (MR) case, presented according to the format in Fig. 2. Cases 1-2 and 4-3 are unstable for both G and GH simulations

**Fig. 7:**
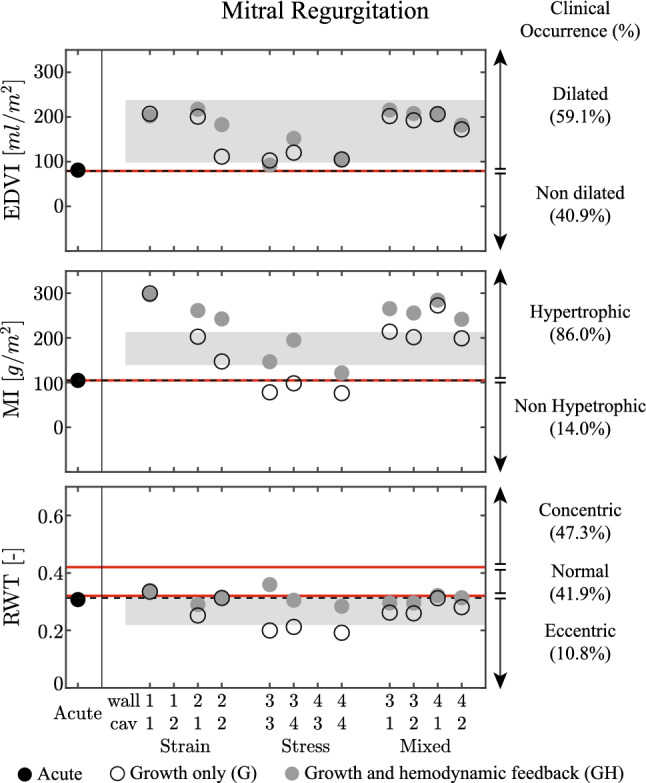
Results for the mitral regurgitation (MR) case, presented according to the format in Fig. 3. On the three panels, patient data are identified as mean with standard deviation, with data collected from Wisenbaugh et al. 1984 for end diastolic volume EDVI and mass index MI, while Seldrum et al. 2018 is used for relative wall thickness RWT. On the right side, patient data are represented as clinical occurrence in percentage (Barbieri et al. 2019a, b)

### Hemodynamic feedback model

The hemodynamic feedback is designed in order to maintain mean arterial pressure (MAP) and cardiac output (CO) at the homeostatic level. To achieve this goal, the peripheral resistance ($R_{\rm {P}}$) and the stressed blood volume ($V_\mathrm{{sb}}$) are updated according to the following differential equations:

13a $$\begin{aligned} \frac{1}{R_{P}} \; \frac{\text {d}R_{P}}{\text {d}t}&\;=\; \frac{S_\mathrm{{CO}}}{\tau _{\rm {hem}}},\quad&\text {with}&\quad S_\mathrm{{CO}} \;=\; \frac{\text {CO} - \text {CO}_\mathrm{{hom}}}{\text {CO}_\mathrm{{hom}}} \end{aligned}$$

13b $$\begin{aligned} \frac{1}{V_\mathrm{{sb}}} \; \frac{\text {d}V_\mathrm{{sb}}}{\text {d}t}&\;=\; -\; \frac{S_\mathrm{{MAP}}}{\tau _{\rm {hem}}},\quad&\text {with}&\quad S_\mathrm{{MAP}} \;=\; \frac{\text {MAP} - \text {MAP}_\mathrm{{hom}}}{\text {MAP}_\mathrm{{hom}}} \end{aligned}$$

where $\tau _{hem}$ is the feedback time constant. The first equation simply expresses that, given a constant MAP, a drop in CO may be compensated for by a drop in $R_{\rm {P}}$ (Eq. 5). The second equation is based on the Frank-Starling law: an increase in $V_\mathrm{{sb}}$ will increase $p_\mathrm{{mc}}$ (Eq. 7) and eventually it will increase the MAP and CO.

### Parameter settings and simulations performed

*Homeostatic state* Settings of the model parameters are based on our previous work (Rondanina and Bovendeerd 2020) and are listed in Table 2. As first step, we simulate a normal cardiac cycle, from which we extract homeostatic settings of the growth stimuli $L_{\sigma ,\text {hom}}$ and $L_{\epsilon ,\text {hom}}$ (Eq. 8) and the hemodynamic feedback control $\hbox {CO}_{{hom}}$ and $\hbox {MAP}_{{hom}}$ (Eq. 13). For all simulations, we consider the cardiac cycle ($T_{\rm {cyc}}$) to last 800 ms. **Acute state.** Second, we introduce three types of valve disease as model perturbation. We simulate AS with a threefold increase of $k_{\rm {AV}}$ during forward flow ($p_{\rm {LV}} > p_{\rm {A}}$) (Eq. 3) (Roger et al. 1997). AR and MR are simulated by a decrease of $k_{\rm {AV}}$ from 10$^{6}$ to 6, when $p_{\rm {LV}} < p_{\rm {A}}$ (Eq. 3) and a decrease of $k_{\rm {MV}}$ from 10$^{6}$ to 30, when $p_{\rm {LV}} < p_{\rm {V}}$ (Eq. 4), to obtain a regurgitant fraction close to 0.6 (Kleaveland et al. 1988; Nakano et al. 1991; Wisenbaugh et al. 1984).

**Table 2:** List of parameters used in the model

Organ		
Parameter	Value	Unit
$C_{\rm {A}}$	20	ml/kPa
$C_{\rm {V}}$	600	ml/kPa
$L_{\rm {A}}$	60	$\hbox {kPa}\,\hbox {ms}^{2}/\hbox {ml}$
$L_{\rm {V}}$	60	$\hbox {kPa}\,\hbox {ms}^{2}/\hbox {ml}$
$R_{\rm {A}}$	10	$\hbox {kPa}\,\hbox {ms}/\hbox {ml}$
$R_{\rm {P}}$	120	$\hbox {kPa}\,\hbox {ms}/\hbox {ml}$
$R_{\rm {V}}$	1	$\hbox {kPa}\,\hbox {ms}/\hbox {ml}$
$T_{\rm {cyc}}$	800	ms
$V_{\rm {A,0}}$	500	ml
$V_{\rm {tot}}$	5000	ml
$V_{\rm {cav,0}}$	67	ml
$V_{\rm {V,0}}$	3000	ml
$V_{\rm {wall}}$	200	ml

The chosen values are adapted from van der Hout-van et al. (2012)

*Growth and hemodynamic feedback* The valve diseases lead the model in a new mechanical loading state in which the growth stimuli of Eq. 8 and hemodynamic stimuli of Eq. 13 are no longer equal to zero. As a result, the cardiac volumes will change according to Eqs. 11 and 12 to restore the myocardial tissue load $L_{\sigma }$ and/or $L_{\epsilon }$ according to the considered growth stimulus (Table 1). In the presence of hemodynamic feedback, the circulatory parameters $R_{\rm {P}}$ and $V_\mathrm{{sb}}$ will also change to recover the hemodynamic function, represented by $\hbox {CO}_{{hom}}$ and $\hbox {MAP}_{{hom}}$, according to Eq. 13. We analyze our results for the case of growth only, indicated by G, and the combination of growth and hemodynamic feedback, indicated by GH.

We assume that cardiac growth, since it requires a volumetric structural change, is a slower process compared with the hemodynamic feedback. For this reason, the constant $\tau _{\rm {grw}}$ is set to 32 ms and $\tau _{\rm {hem}}$ 16 ms, making the hemodynamic feedback twice as fast as the cardiac growth.

### Model evaluation

We quantify cardiac growth with the LV end diastolic volume index (EDVI), the LV mass index (MI) and the relative wall thickness (RWT). The EDVI and MI are defined as the end diastolic volume ($V^{\text {max}}_{\rm {cav}}$) and LV mass divided by the body surface area, which is set to 2 $\text {m}^{2}$ (Lang et al. 2015), while RWT is computed as ratio between wall thickness and cavity radius both at end diastole. Following the classification proposed by Gaasch and Zile 2011, we identify dilated configurations, having EDVI higher than 79 ml/$\hbox {m}^{2}$, and hypertrophic cases, with MI higher than 105 g/$\hbox {m}^{2}$. Moreover, we identify the geometry as eccentric if RWT is lower than 0.32, normal if RWT is between 0.32 and 0.42, and concentric if RWT is higher than 0.42.

To evaluate the models, we compare simulations results with clinical data. Data obtained from Guzzetti et al. (2019), Seldrum et al. (2018), Wisenbaugh et al. (1984) are presented by the mean and standard deviation of the cardiac indexes EDVI, MI and RWT, as shown in the left panels of Figs. 3, 5 and 7. Data from (Barbieri et al. 2019a, b) are presented in terms of clinical occurrence, see the right panels of the same figures.

## Results

As we adopted model parameter settings for the healthy state from (Rondanina and Bovendeerd 2020), we find the same homeostatic state identified by a cardiac output ($\hbox {CO}_{{hom}}$) of 5.2 l/min and a mean arterial pressure ($\hbox {MAP}_{{hom}}$) of 12.2 kPa. Maximum and minimum cavity volume ($V_{\rm {cav}}^{\text {max}}$ and $V_{\rm {cav}}^{\text {min}}$) are 154 ml and 87 ml, respectively, and a maximum LV pressure ($p_{\rm {cav}}^{\text {max}}$) is 18.2 kPa. These values lead to a homeostatic state characterized by local tissue loads of $L_{\sigma ,\text {hom}}^\mathrm{{avg}}$ of 19.2 kPa, $L_{\sigma ,\text {hom}}^\mathrm{{max}}$ of 59.3 kPa, $L_{\epsilon ,\text {hom}}^\mathrm{{amp}}$ of 0.12 and $L_{\epsilon ,\text {hom}}^\mathrm{{max}}$ of 0.17.

### Aortic stenosis

*Acute state* In the acute state AS leads to a decrease in MAP and CO around -20% as shown in Fig. (2). Despite the decrease in MAP, $p_{\rm {cav}}^{\text {max}}$ is increased to 21.3 kPa, due to the increased pressure drop over the stenotic valve. At tissue level, this increase is reflected in a positive value of both stress stimuli. $V_{\rm {cav}}^{\text {max}}$ remains about the same at 163 ml, but $V_{\rm {cav}}^{\text {min}}$ increases to 108 ml, causing $L_{\epsilon }^\mathrm{{max}}$ to remain close to zero, but $L_{\epsilon }^\mathrm{{amp}}$ to decrease.

*Growth only cases* With growth only and no hemodynamic feedback, indicated by G in Fig. (2), the strain-only case 1-2, with $L_{\epsilon }^\mathrm{{amp}}$ driving wall growth and $L_{\epsilon }^\mathrm{{max}}$ driving cavity growth (see Table 1), displays a decrease of $V_{\rm {wall}}$ towards zero and an unbounded increase of $V_{\rm {cav,0}}$. For these volumes, the model of hemodynamics could not be solved. The other cases show stable growth, where the controlled strain measure is fully restored and the remaining stress and strain stimuli are decreased with respect to their values in the acute case. In the stress-only cases, model 4-3, with $L_{\sigma }^\mathrm{{max}}$ driving wall growth and $L_{\sigma }^\mathrm{{avg}}$ driving cavity growth, did not yield stable growth, mostly due to an unbounded increase of $V_{cav,0}$. The other cases show stable growth, where the controlled stress measure is fully restored and the remaining stress and strain stimuli are small. In the mixed cases, the controlled $L_{\epsilon }$ and $L_{\sigma }$ are restored to their homeostatic levels, while the other stimuli tend to be reduced as well. LV wall volume decreases in most of the 10 stable cases, while the cavity volume decreases, except for the strain-only case with case 2-2.

*Growth and hemodynamic feedback* While local tissue load is restored in the other growth only cases, according to the controlled stimulus, LV hemodynamic function is not. Adding hemodynamic feedback, as indicated by GH in Fig. 2, leads to restoration of hemodynamic function in all 10 stable cases, identified by $S_\mathrm{{CO}}=S_\mathrm{{MAP}}=0$ (Fig. 2, right panel). The hemodynamic feedback does not solve the instabilities in the growth only models 1-2 and 4-3. For case 1-2, the influence of the hemodynamic feedback is not significant. For case 4-3, the change in hemodynamic parameters (peripheral resistance $R_{\rm {P}}$ and stressed blood volume $V_\mathrm{{sb}}$) allows to simulate more growth steps, but both cavity and wall volume display unbounded growth eventually. In the strain-only case 1-1, this is achieved by large changes (more than 50%) in hemodynamic parameters. For all the remaining cases, the changes are within 20%. Regarding the cardiac volumetric change ($V_{\rm {wall}}$ and $V_{\rm {cav,0}}$), the strain-only case with case 2-2 converges at an increase of 300% for both volumes. For all the remaining cases, cavity volume decreases while wall volume increases, with changes being below 50%. Eventually adding hemodynamic feedback tends to increase the non-controlled stress and strain stimuli in strain-driven growth. For the stress-driven and mixed cases, non-controlled stimuli remain fairly constant.

*Comparison with clinical data* In Fig. 3, we compare model output with clinical data. The left panels show how clinical data are characterized by a decrease in end diastolic volume index EDVI and an increase in relative wall thickness RWT, while left ventricular mass index MI shows no significant change (Guzzetti et al. 2019). EDVI and MI are predicted fairly well in all simulations, except for the strain-based model 2-2. RWT is generally underestimated without hemodynamic feedback, but improves when adding it. Strain-based models 1-1 and 2-2 do not yield realistic results for RWT. The right side of Fig. 3 show clinical data on EDVI, MI and RWT in terms of prevalence in the patient population (Barbieri et al. 2019a). It shows that growth upon AS is most clearly apparent in MI and RWT, while not reflected at all in EDVI. Again observations on RWT are captured best by the stress-only and mixed models along with the strain only model for case 2-1, especially with the addition of hemodynamic feedback.

### Aortic regurgitation

*Acute State* In the acute case, AR leads toward a decrease in MAP and CO around 20% (Fig. 4, right). The regurgitant valve causes an increase of $V_{\rm {cav}}^{\text {max}}$ to 180 ml, while $V_{\rm {cav}}^{\text {min}}$ decreases to 83 ml causing both strain stimuli to increase. The minor drop in $p_{\rm {cav}}^{\text {max}}$ to 17 kPa causes both stress stimuli to remain close to zero (Fig. 4, left panel).

*Growth only cases* With strain-only feedback, the case 1-2 does not converge due to a decrease of $V_{\rm {wall}}$ toward zero and an unbounded increase of $V_{\rm {cav,0}}$. In the cases driven by one stimulus only (1-1 and 2-2), the non-controlled stimuli tend to increase. Case 2-1 causes all stimuli to approach zero. In the stress-only feedback, the case 4-3 does not converge due to a decrease of LV volumes toward zero. The remaining cases show stable growth, with the controlled stress measure fully recovered while the strain stimuli remain almost unchanged compared with the acute state. In the mixed cases, the controlled stimuli return to zero, while the others are close to zero. Strain-only and mixed cases have an increase in $V_{\rm {wall}}$ and $V_{\rm {cav,0}}$ while with the stress-only cases we do not obtain significant changes, except with case 3-4 which is also characterized by an increased $V_{\rm {wall}}$ and $V_{\rm {cav,0}}$. In Fig. (4,right panel), we see how the strain-only case 2-1 and the mixed cases have a recovered hemodynamic function. In the other models, hemodynamic function is still decreased.

*Growth and hemodynamic feedback* Hemodynamic function is restored in all stable growth cases upon adding hemodynamic feedback. The hemodynamic feedback however does not solve the instabilities in the growth only models 1-2 and 4-3, which are characterized by a similar divergence as observed for the growth only cases. In the cases where hemodynamic function was restored already in the growth-only cases, changes in circulatory parameters $R_{\rm {P}}$ and $V_\mathrm{{sb}}$ are about zero. Cases 2-2 and 3-4, that already showed improvement in hemodynamic function in the growth-only situation, require small changes in $R_{\rm {P}}$ and $V_\mathrm{{sb}}$. The remaining cases 1-1, 3-3 and 4-4 require changes in$R_{\rm {P}}$ and $V_\mathrm{{sb}}$ of 15–30%. Regarding LV volumetric growth, we observe an increase in $V_{\rm {wall}}$ and $V_{\rm {cav,0}}$ for all 10 stable cases, except for a decrease in $V_{\rm {cav,0}}$ obtained with the stress-only stimuli for cases 3-3 and 4-4.

*Comparison with clinical data* The left panel of Fig. 5 shows that clinical data are characterized by an increase in end diastolic volume index EDVI and left ventricular mass index MI, with a decrease of the relative wall thickness RWT (Seldrum et al. 2018; Wisenbaugh et al. 1984). In the strain-only models, these observations are best captured in case 1-1. In the stress only models, adding hemodynamic feedback improves the results for MI but worsens those for RWT, while the EDVI remains almost unchanged. The mixed models show good agreement for EDVI, but overestimate MI and fail to predict the decrease in RWT. The right side of Fig. 5 shows clinical data on EDVI, MI and RWT in terms of prevalence in the patient population (Barbieri et al. 2019b). It shows that result of all growth models agree with the clinical observations that EDVI and MI are increased, indicating dilated hypertrophic hearts. As RWT shows no significant clinical pattern, it cannot be used to judge the quality of the growth models.

### Mitral regurgitation

*Acute state* In the acute state, MR leads to a decrease in MAP and CO of about 20%, as shown in Fig. 6. The backflow through the mitral valve causes a decrease in $p_{\rm {cav}}^{\text {max}}$ at 15 kPa and $V_{\rm {cav}}^{\text {min}}$ at 56 ml, causing negative stress stimuli and a positive $L_{\epsilon }^\mathrm{{amp}}$. Since $V_{\rm {cav}}^{\text {max}}$ remains approximately the same, $L_{\epsilon }^\mathrm{{max}}$ is about zero.

*Growth only cases* The strain-only case 1-2 does not converge because of a steep increase of $V_{\rm {wall}}$. The other strain-only cases show stable growth, with the controlled strain measure fully recovered. The remaining strain and stress stimuli are close to the homeostatic level for cases 1-1 and 2-1, but remain unchanged for case 2-2. In the stress-only cases, model 4-3 did not yield stable growth due to a decrease of both volumes towards zero. The remaining cases show stable growth, with the controlled stress measure fully recovered and the remaining stress stimulus close to homeostatic level, while all remaining strain stimuli are positive. Regarding the mixed cases, the controlled stimuli are fully recovered with the remaining stimuli close to the homeostatic state. With the stress-only models, we observe a general decrease of $V_{\rm {wall}}$ and an increase of $V_{\rm {cav,0}}$, while for the strain-only and mixed cases we find an increase of both volumes.

*Growth and hemodynamic feedback* The hemodynamic feedback however does not solve the instabilities in the growth only models 1-2 and 4-3. These are characterized by a similar change in volume as in the growth only cases. Restoration of MAP and CO through hemodynamic feedback (GH) requires changes in $R_{\rm {P}}$ and $V_\mathrm{{sb}}$ below 3% for the strain-only and mixed cases, and more pronounced changes in the stress-only case, reaching up to almost 50% for case 3-3 and 4-4. Adding hemodynamic feedback causes an increase of $V_{\rm {wall}}$ in most converged cases, except for case 1-1. $V_{\rm {cav,0}}$ increases with case 2-2 and 3-4, decreases with 3-3 and 4-4, while it remains almost unchanged for the remaining cases.

*Comparison with clinical data* Clinical data in the left panels of Fig. 7 show an increase in end diastolic volume index EDVI and mass index MI, while the relative wall thickness RWT tends to decrease (Seldrum et al. 2018). The observations on EDVI are captured by all the 10 converged simulations. For MI, the addition of hemodynamic feedback helps only the stress models 3-3 and 3-4, while it causes an over-estimation for the strain-only and mixed models. The right side of Fig. 7 shows clinical data on EDVI, MI and RWT in terms of prevalence in the patient population (Barbieri et al. 2019a, b). It shows that growth upon MR is most clearly apparent in MI and EDVI. For these cases, adding the hemodynamic feedback improves the results.

## Discussion

Cardiac growth is one of the adaptation for the heart to respond mechanisms to changes in preload and afterload. In a previous study, (Rondanina and Bovendeerd 2020) we simulated growth in response to valve disease for several combinations of stress and strain based stimuli. In most cases, we observed a decrease in hemodynamic function, expressed in terms of mean arterial pressure (MAP) and cardiac output (CO), between 20 and 40%. In the current study, we evaluate the hypothesis that such a decrease is counteracted by an adaptive response of the circulatory system.

### Considerations on the methods

Hemodynamic regulation is a complex process which involves short- and long-term mechanisms to maintain blood supply and consequently oxygen delivery at an adequate level. It involves hormone synthesis along with the activity of the sympathetic nervous system (Cowley Jr 1992; Dampney et al. 2002; Guyenet 2006; Hall 2015). There is evidence in literature that both MAP and CO are regulated by an adaptation of vasculature resistance $R_{\rm {P}}$ and blood volume $V_\mathrm{{sb}}$ (Cowley Jr 1992; Guyton 1967, 1981; Jacobsohn et al. 1997).

In our hemodynamic regulation model, we indeed control MAP and CO, through changes in $R_{\rm {P}}$ and $V_\mathrm{{sb}}$, but do not aim for a detailed description of the influence of the nervous system.

Regarding the speed of growth and hemodynamic feedback (Eqs. 11 and 13), we reasoned that the body shall react first to a change in hemodynamic load with the hormonal and neural response causing vasodilation or vasoconstriction of the peripheral arteries, and hence $R_{\rm {P}}$, or changes in renal function, affecting $V_\mathrm{{sb}}$. Cardiac growth would occur at a longer timescale in case of a persisting change in load. For this reason, our hemodynamic feedback constant $\tau _{\rm {hem}}$ is smaller than the growth constant $\tau _{\rm {grw}}$. The actual values are chosen in order to limit simulation times. Obviously, the real timescale would be much longer, presumably on the order of months. As shown in our previous work (Rondanina and Bovendeerd 2020), these constants might affect the time course of changes in circulatory and cardiac parameters, but they do not interfere with the final ending state of the model. We verified this by varying the ratio $\tau _{grw}/\tau _{hem}$ over a range $1/16 \le \tau _{grw}/\tau _{hem} \le 16$.

We employ a phenomenological growth law, which is common in many growth models (Witzenburg and Holmes 2017). Such models assume that fiber stress or strain (or both) can be sensed by cardiomyocytes, and that these cells respond by growth along or perpendicular to the fiber direction. They do not address the actual processes at (sub-)cellular level. The simplification at this level makes it computationally feasible to evaluate the effect of growth at organ level and to even include adaptation of the circulatory system. In comparison with finite element (FE) models, our model lacks the ability to describe spatially varying growth in response to spatially varying changes in myocardial load, as induced for example by myocardial infarction or conduction disorders. As an advantage, we avoid the numerical problems that may arise in FE models, typically related to distortion of elements during growth or uncertainty on boundary conditions (van Osta et al. 2019). Thus we are better able to test the intrinsic stability of a potential growth law. In addition, the computational load of our model is orders of magnitude less than that of FE models, allowing a quick evaluation of different types of growth laws, and offering more potential for eventual use in the clinic. It has not been established yet what is the most representative stimulus for cardiac growth. In the literature, several models have been proposed with a growth law based on a single stimulus (Kroon et al. 2009) or on multiple stimuli (Arts et al. 1994, 2005; Kerckhoffs et al. 2012b; Taber 1998). In general, these stimuli are either stress-based or strain-based (Bovendeerd 2012; Witzenburg and Holmes 2017), although a mixed stress–strain stimulus has been used as well (Taber and Chabert 2002). Often growth is driven by a stress stimulus upon pressure overload and a strain stimulus during volume overload (Göktepe et al. 2010). Also in our model, we investigate a mixed stress–strain stimulus. We note that stress and strain are linked through constitutive equations, but that the equation for active stress is time dependent. Hence, a full recovery of stress or strain to the homeostatic state does not necessarily imply a recovery of the counterpart strain or stress. As a consequence, a complete recovery of the strain level does not necessarily mean a recovery of the stress level.

### Considerations on the results

The growth only cases in general cause a decrease in hemodynamic function identical to the one found in our previous study (Rondanina and Bovendeerd 2020). The addition of the hemodynamic feedback caused hemodynamic function to be restored to its homeostatic level in all 10 stable stimuli combinations out of the 12 combinations tested. To assess whether the changes in $R_{\rm {P}}$ and $V_\mathrm{{sb}}$ are realistic, we first address clinical observations. The reported range of $R_{\rm {P}}$ for control cases is between $134.6 \pm 29.9$ kPa ms/ml and $169.5 \pm 34.5$ kPa ms/ml (Ganau et al. 1992; Huang et al. 2011; Remmen et al. 2005). For AS, it is between $118.2 \pm 14.3$ kPa ms/ml and $194.2 \pm 60.3$ (Friedrich et al. 1994; Lloyd et al. 2017; Rajani et al. 2010). For AR, it is between $126.4 \pm 11.2$ kPa ms/ml and $169.5 \pm 29.8$ kPa ms/ml. Finally, for MR it is between $147.0 \pm 31.0$ kPa ms/ml and $159.0 \pm 34.0$ kPa ms/ml. These data suggest that $R_{\rm {P}}$ stays within the normal range for the various valve pathologies. As indicator of $V_\mathrm{{sb}}$, we can use the mean circulatory pressure $p_\mathrm{{mc}}$ (Eq. 7), that has a normal value of $2.93 \pm 1.07$ kPa (Lorsomradee et al. 2007). In this case, we observe a general increase of $p_\mathrm{{mc}}$ for AS (Carroll et al. 1992; Lloyd et al. 2017; Martinez et al. 2012) and MR (Kainuma et al. 2011), with values from $2.93 \pm 0.93$ kPa to $5.33 \pm 1.33$ kPa, but not for AR (Greenberg et al. 1981; Lorsomradee et al. 2007), which values span from $2.53 \pm 0.53$ kPa to $2.93 \pm 0.67$ kPa.

With respect to the change in cardiac indexes EDVI, MI and RWT, we note that the clinical data considered for Figs. 3, 5 and 7 are in general agreement. Differences occur with respect to EDVI and MI for AS, as well as the RWT for the MR and AR. These differences might be caused by the presence of simultaneous moderate diseases in (Barbieri et al. 2019a, b) which led to secondary effects. Due to a lack of clinical occurrence data for a severe MR, in Fig.  7 both (Barbieri et al. 2019a) and (Barbieri et al. 2019b) are considered. The resulting clinical occurrence refers to moderate MR cases in presence of a severe AS or AR.

With the strain-based growth laws, case 2-1 performed best. In line with experimental observations, changes in $R_{\rm {P}}$ and $V_\mathrm{{sb}}$ are small. Cardiac indexes EDVI, MI and RWT are predicted well, except for an overestimation of MI in MR. Case 2-2 yields small changes in $R_{\rm {P}}$ and $V_\mathrm{{sb}}$ as well, but EDVI and MI are overestimated in both AS and AR. Case 1-1 requires unrealistically large changes in $R_{\rm {P}}$ and $V_\mathrm{{sb}}$ in AS and AR, whereas MI is severely overestimated in MR. Finally, case 1-2 did not converge at all.

For the stress-based growth laws, case 3-4 performed best. Changes in $R_{\rm {P}}$ and $V_\mathrm{{sb}}$ are small and cardiac indexes are predicted well, except for a large RWT in AR. Cases 3-3 and 4-4 show unrealistic changes in $R_{\rm {P}}$ and $V_\mathrm{{sb}}$ during AR and MR. Finally, case 4-3 did not converge at all.

For the mixed stress-strain cases, we first note that the final state for the LV and the circulation is independent of the way the growth stimuli are applied, as was also observed in our previous study. The results of all mixed simulations are similar. Changes in $R_{\rm {P}}$ and $V_\mathrm{{sb}}$ are small, in line with experimental observations. Also changes in cardiac indexes match experimental observations, except for an overestimation of RWT and MI in AR, and an overestimation of MI in MR. In this respect, adding hemodynamic feedback improved prediction of RWT in AS, but worsened prediction of MI in MR. Still, the overall affect of adding hemodynamic feedback in the mixed models is positive, as it restores hemodynamic function to normal, physiologically realistic levels, in particular in the AS and MR scenarios.

Since the mixed models are less dependent on the precise nature of the stimulus and because the true nature of the growth stimulus is not known yet, we think that these models are most promising for future research. We note that the comparison of model results with clinical data is not trivial. The amount of change in cardiac indexes and hemodynamic parameters obviously depends on the severity of the disease. We model the AS through a threefold increase of aortic resistance, while AR and MR are characterized by a regurgitant fraction close to 0.6. We verified that a different level of severity did not affect the type of hypertrophy, even though it leads toward a different ending state. While the isolated perturbation in the model facilitates our analysis, at the same time it might not be representative for real clinical cases, where the valve disease might progress and secondary pathologies might play a role.

### Comparison with other models

In the literature, the majority of the studies on modeling growth focus on LV geometry but pay less attention to the circulation. Arts et al. (2005) proposed a model of hemodynamic control in which the blood volume and the peripheral pulmonary resistance were adapted to simulate pressure control. Moreover, the geometry of the vessels was also changed to sustain changes in blood flow. Later, Kerckhoffs et al. (2012a) adopted this model to simulate a left bundle branch block, in which also the cardiac output was regulated by peripheral resistance. Along with these parameters, other candidates for the hemodynamic feedback are the arterial and venous compliance ($C_{A}$ and $C_{V}$), the LV elastance and the heart rate (Beard et al. 2013; Witzenburg and Holmes 2019). Regarding the heart rate, we maintain this parameter constant. We hypothesize that a change of heart rate might be interpreted as an incomplete hemodynamic adaptation rather than a direct consequence of the studied disease. Moreover, in literature we did not find any significant correlation between heart rate and valve disease (Akinboboye et al. 2004; Seldrum et al. 2018). Eventually updates in $C_{A}$ and $C_{V}$ affect cardiac function in a similar manner as an update in $V_\mathrm{{sb}}$: They change the mean circulatory filling pressure (Eq. 7) and affect cardiac function through the Frank-Starling effect.

Our analysis is similar to the one proposed by Witzenburg and Holmes (2018) for AS and MR. These authors also combined lumped parameters models of left ventricular and circulatory mechanics with a phenomenologic growth law. They fitted circulatory and growth law parameters to match results from hemodynamic overload studies in dogs and tested to what extent the resulting model predicted growth in independent studies of hemodynamic overload. They describe LV mechanics with a time-varying elastance model, that does not allow for an easy relation between constitutive properties at organ level (describing pressure-volume relations through compartmental parameters ‘A,’ ‘B,’ ‘E’ and ‘$V_{0}$’) and tissue level (describing stress-strain relations through material parameters ‘a,’ ‘b’ and ‘e’). This relation occurs more naturally in the one-fiber model that we use in our study, as shown in Eq. 1a. Consequently, growth-induced changes in cavity and wall volume are also reflected in the LV pressure–volume behavior more naturally. This model also enables computation of local tissue load, with the limitation that fiber stress and strain should be considered as representative spatially averaged values. Hence, it is possible to establish a natural stimulus–effect relation, from tissue load to change in cardiac size.

Considering the circulatory system, Witzenburg and Holmes (2018) match acute hemodynamic data from the experiments and prescribe the evolution of resistance $R_{\rm {P}}$ and the degree of mitral valve regurgitation. In our approach, we prescribe a constant valve pathology and adapt $R_{\rm {P}}$ and $V_\mathrm{{sb}}$ according to our hemodynamic feedback model. Interestingly, Witzenburg and Holmes (2018) find that matching acute changes in hemodynamics is more important than matching the subsequent evolution, suggesting that this evolution involves minor changes as compared to the acute changes. This observation matches with clinical data and supports our selection of the most promising models on the basis of minor changes in $R_{\rm {P}}$ and $V_\mathrm{{sb}}$.

Considering the growth law, Witzenburg and Holmes (2018) investigate one option, considered most promising in an earlier study (Witzenburg and Holmes 2017). In this model, an increase in maximum circumferential strain results in an increase in cavity volume and an increase in maximum radial strain results in an increase in wall volume. In our model, we do not consider maximum radial strain, or its surrogate, minimum fiber strain. The option resembling the one in Witzenburg and Holmes (2018) best would be the strain-based model 2-1 with maximum fiber strain driving wall growth and strain amplitude driving cavity growth. Indeed, we find that this model performs well in the case of AS and MR, investigated by Witzenburg and Holmes (2018). However, our models with a mixed stimulus perform equally well. This confirms the more general conclusion of Witzenburg and Holmes (2017), that the most promising growth laws employ multiple inputs.

### Limitations and outlook

An important limitation of our study is that we considered two strain stimuli and two stress stimuli only. It would be interesting to extend the analysis to more stimuli. For example, minimum sarcomere length could be used as an alternative strain stimulus, to enable better comparison with the study of Witzenburg and Holmes (2018). Our analysis could also be extended to other cardiac conditions, for example the growth of the athlete’s heart where presumably cardiac growth occurs homogeneously throughout the wall. As addressed above, to assess growth in conditions that involve spatially varying changes in tissue load, the step towards a finite element model should be made. The findings of our current study might be used to guide the choice of the growth model in the finite element model. Finally, the current model may already form a basis for a tool to predict patient-specific growth in response to spatially homogeneous changes in tissue load, since it is computationally inexpensive. As a first step towards this goal, the model should be tested on its ability to predict growth in individual rather than generic cases, similar to the approach followed by Witzenburg and Holmes (2019).

Finally, we focused on growth models that resulted in a stable ending state. While a stable state may be expected to exist clinically for minor valve pathologies, it is unclear whether it would exist for the degree of valve dysfunction used in our simulations. Such data are unavailable since, in the clinical case, potential unbound growth would probably be prevented by valve replacement.

Despite these considerations we think the proposed analysis still offers valuable points of reflection.

### Conclusion

We investigated cardiac growth and circulatory adaptation in response to three valve diseases (aortic stenosis, aortic regurgitation and mitral regurgitation). We integrated a lumped multiscale model of LV mechanics and a lumped model of circulatory hemodynamics with a model for tissue growth and hemodynamic feedback. Our study shows the importance of coupling growth with hemodynamic feedback. With our model, we succeeded in restoring the homeostatic state at circulatory level, characterized by pressure and flow, and at tissue level, expressed in various combinations of stress and strain. The results obtained by using a combination of stress and strain stimuli to drive cardiac growth (1) matched clinical observations on cardiac growth well, (2) required only a small, clinically realistic adaptation of the properties of the circulatory system and (3) were fairly insensitive to the exact choice of the chosen mechanics loading measure. Thus, this study suggests to model cardiac growth using a mixed stress-strain stimulus as input, to maintain homoeostatic tissue load, in combination with a model of hemodynamic feedback to maintain cardiac pump function.
